# Fracture types affect clinical outcomes of patients managed within the fracture liaison and osteoporosis medication management services

**DOI:** 10.1038/s41598-019-46315-4

**Published:** 2019-07-12

**Authors:** Chirn-Bin Chang, Rong-Sen Yang, Lo-Yu Chang, Jen-Kuei Peng, Keh-Sung Tsai, Wei-Jia Huang, Tsung-Han Yang, Ding-Cheng Chan

**Affiliations:** 1 Department of Geriatrics and Gerontology, National Taiwan University Hospital, Taipei, Taiwan; 2 Department of Internal Medicine, Chu-Tung Branch, National Taiwan University Hospital, Hsinchu County, Taiwan; 3 Department of Internal Medicine, National Taiwan University Hospital, Taipei, Taiwan; 4 Department of Orthopaedics, National Taiwan University Hospital, Taipei, Taiwan; 5 College of Medicine, National Taiwan University, Taipei, Taiwan; 6 Department of Family Medicine, Bei-Hu Branch, National Taiwan University Hospital, Taipei, Taiwan; 7 Superintendent Office, Far Eastern Polyclinic of Far Eastern Medical Foundation, Taipei, Taiwan; 8 Department of Orthopaedics, Hsinchu Branch, National Taiwan University Hospital, Hsinchu City, Taiwan; 9 Superintendent Office, Chu-Tung Branch, National Taiwan University Hospital, Hsinchu County, Taiwan

**Keywords:** Fracture repair, Geriatrics

## Abstract

Osteoporosis medication in fragility fracture patients is associated with better outcomes. However, limited studies have investigated whether fracture types affect outcomes among patients undergoing treatment. We performed a secondary data analysis on participants from a fracture liaison service and an osteoporosis medication management service. Participants (n = 974) were regrouped into hip fracture (HF), vertebral fracture (VF), HF + VF, and NO HF/VF groups at baseline. Bivariate and multivariate logistic regressions were performed to identify baseline correlates on one-year mortality, incident refractures, and falls. Baseline characteristics were different among fracture groups. The HF group was oldest, with the lowest body mass index (BMI), lowest FRAX® T-score and had the highest 10-year fracture risk. After intervention, the HF group still had the highest mortality, but the HF + VF group had the highest refracture and incident fall rates. In the multivariate regression analysis, prevalent HF and VF, lower BMI and albumin level, and having chronic kidney disease or cancer were associated with higher mortality rates. HF + VF patients had the highest refracture risk. Prevalent HF and VF, older age and higher BMI, and having cancer or osteoarthritis were associated with a greater fall risk. HF and VF are associated with adverse outcomes, even under an optimal fracture care.

## Introduction

Osteoporotic fractures are associated with higher mortality rates, subsequent fractures, healthcare costs and lower function and quality of life. It has been projected that the number of osteoporotic fractures is expected to increase worldwide^1,2^ and will cause a disproportionally high burden on the healthcare system in near feature^2,3^. Osteoporosis, with a prevalence of 27% among older Taiwanese, is ranked the 3^rd^ most common health problem with the highest hip fracture (HF) incidence in the Asian-pacific region^4^. However, having a lower awareness and a lower rate of receiving bone mineral density (BMD) tests and osteoporosis medications than other countries were also found^5^. Optimized comprehensive care should be provided for older adults with a high risk of osteoporotic fracture.

Fracture liaison services (FLSs) are developed in many countries with specified protocols that include identification of patients with fragility fracture, a series of investigations and risk assessments for osteoporosis, and education to prevent osteoporosis and fractures. More importantly, appropriate medication treatments for osteoporosis were initiated with monitoring of compliance and adherence^6^. Since 2014, National Taiwan University Hospital (NTUH) Main Hospital (MH) and its affiliated Bei-Hu Branch (BB) created FLSs as secondary fracture prevention programs (SFPs) for those with osteoporosis/fragility fracture and a complementary program named the “medication management service (MMS)” to expand the care on those patients who need osteoporosis medication monitoring but do not necessarily have fragility fracture^7^. Some studies have described that being enrolled in an FLS reduced the subsequent fracture rate and improved mortality^8–10^ compared with usual care while cooperating with effective osteoporosis treatments^11^. From previous studies, predictors of subsequent fractures among treatment-naïve patients with fracture have been demonstrated, such as women aged 85 years or older^12^. Excess mortality was highest among patients with HFs and lowest for those with minor fractures^13,14^. Nonetheless, the parameters to predict subsequent fractures, incident falls, and mortality among those enrolled in the optimal osteoporosis management program are limited. To identify patients with a risk of adverse outcomes despite optimal care, we primarily aim to investigate whether fracture types or other participant characteristics contribute to subsequent fractures, incident falls, and mortality among participants within the FLS and MMS programs. The secondary aim is to investigate whether lifestyles such as exercise habits, use of calcium and vitamin D_3_ supplementation, and adequate protein intake are improved after these programs.

## Results

### Study population

**Figure 1:**
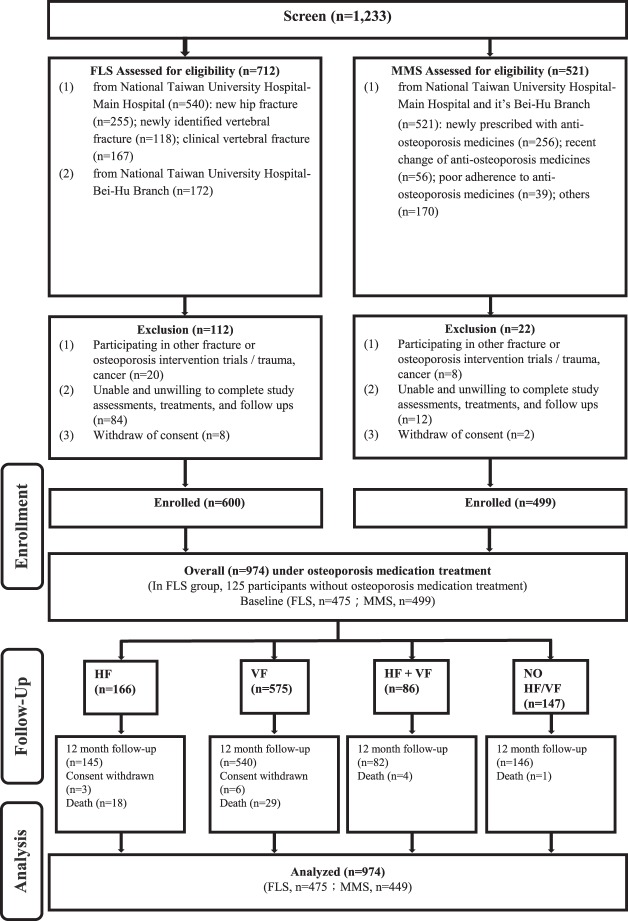
Study flow chart. Participants were excluded from the service after screening if (1) the fractures were related to trauma, cancer, or atypical fracture at femoral shaft; (2) participating physicians felt that the patients’ life expectancy are ≦2 years; (3) unable and unwilling to complete study assessments and follow-up; (4) participating in other fracture or osteoporosis intervention trials. After enrollment, interventions were performed and they were followed for 12 months. Participants with osteoporosis medications (n = 475 in FLS group and n = 499 in MMS group) are regrouped into HF, VF, HF + VF and NO HF/VF for secondary data analysis. HF: participants with hip fracture, VF: participants with vertebral fracture. HF + VF: participants with hip fracture and vertebral fracture. NO HF/VF: participants with no hip fracture or vertebral fracture.

In total 1233 participants were screened, 1199 entered FLS and MMS, and 974 (79%) participants under osteoporosis medication treatment were analyzed in the current study (Fig. 1). The hip fracture (HF) group had 166 participants, and the vertebral fracture (VF), hip plus vertebral fracture (HF + VF), and no hip or vertebral fracture (NO HF/VF) groups had 575, 86, and 147 participants, respectively. The follow-up rate was highest as 99.32% for NO HF/VF group but lowest as 87.35% for HF group. The other two groups have follow-up rate higher than 90% (Fig. 1).

### Baseline characteristics

Table 1 shows the baseline characteristics of the overall participants and differences among the 4 groups. There were significant differences of baseline characteristic among them. For the factors influencing primary outcomes, including age, sex, body mass index (BMI), secondary osteoporosis, and FRAX® score (10-year major osteoporotic fracture [MOF] risk and HF risk), the HF group was oldest, had the lowest percentage of females, lowest BMI, lowest rates of secondary osteoporosis, highest FRAX® score, highest 10-year risk of MOF and highest risk of HF. The VF group had the highest BMI, and the NO HF/VF group was the youngest among the 4 groups. These four groups had a similar proportion of participants with a history of smoking, parents with HFs and rheumatoid arthritis, and use of steroids. For laboratory data, the HF group had the lowest albumin and calcium levels, but the NO HF/VF group had the highest calcium and albumin levels. The HF + VF group had the highest alkaline phosphatase (ALP) level. Significant between-group differences among comorbidities were apparent in the prevalence of hypertension, diabetes mellitus, heart disease, thyroid and parathyroid gland disease, and neurological disease. In the post hoc analysis, the VF group had a higher proportion of participants with hypertension, diabetes mellitus, and heart disease than did the NO HF/VF group.

**Table 1:** Baseline characteristics of the overall participants and differences among the 4 groups.

	Total	HF	VF	HF + VF	NO HF/VF	P-value
**Basic demographic variables**						
N	974	166	575	86	147	
Age (years)	76.1 ± 10.2	79.0 ± 9.8^c^	77.3 ± 9.6^e^	76.2 ± 11.0^f^	68.5 ± 9.4	<**0**.**001**
Sex (female)	788 (80.9%)	122 (73.5%)^bc^	457 (79.5%)^e^	75 (87.2%)	134 (91.2%)	<**0**.**001**
**FRAX-related variables**						
Weight (kg)	54.2 ± 9.9	53.8 ± 10.4	54.9 ± 10.3^e^	53.3 ± 8.5	52.4 ± 7.9	**0**.**04**
Height (cm)	154.2 ± 7.8	156.7 ± 7.7^abc^	153.6 ± 8.2	152.9 ± 7.0	154.4 ± 6.3	<**0**.**001**
BMI (kg/m^2^)	22.8 ± 3.8	21.8 ± 3.6^ab^	23.2 ± 3.9^e^	22.9 ± 3.6	22.0 ± 3.3	<**0**.**001**
History of fracture	808 (83.0%)	166 (100%)^abc^	555 (96.5%)^e^	77 (89.5%)	10 (6.8%)	<**0**.**001**
Parents hip fracture	75 (7.8%)	16 (9.6%)	40 (7.0%)	3 (3.5%)	16 (10.9%)	0.14
Smoke	20 (2.1%)	7 (4.2%)	9 (1.6%)	3 (3.5%)	1 (0.7%)	0.08
Steroid use	34 (3.5%)	3 (1.8%)	23 (4.0%)	4 (4.7%)	4 (2.7%)	0.49
Rheumatoid arthritis	19 (2.0%)	2 (1.2%)	11 (1.9%)	3 (3.5%)	3 (2.0%)	0.67
Secondary osteoporosis	94 (9.7%)	11 (6.6%)^c^	49 (8.5%)^e^	8 (9.3%)	26 (17.7%)	**0**.**04**
Alcohol intake >3 units/d	11 (1.1%)	1 (0.6%)	8 (1.4%)	2 (2.3%)	0 (0%)	0.32
FRAX® T-score	−2.81 ± 0.86	−3.14 ± 0.67^ac^	−2.72 ± 0.85	−2.93 ± 1.29	−2.68 ± 0.67	<**0**.**001**
10-yr major osteoporotic fracture risk (with BMD)	22.5 ± 11.5	25.3 ± 11.8^c^	22.5 ± 10.6^e^	24.0 ± 11.4^f^	15.1 ± 8.5	<**0**.**001**
10-yr hip fracture risk (with BMD)	11.7 ± 9.6	14.3 ± 10.5^ac^	11.2 ± 8.4^e^	12.2 ± 7.7^f^	7.2 ± 7.2	<**0**.**001**
**Health-related variables**						
Alkaline phosphatase (U/L)	80.0 ± 44.8	90.2 ± 41.2^a^	74.5 ± 37.9^d^	107.1 ± 84.4^f^	69.1 ± 43.6	<**0**.**001**
Calcium (mmol/L)	2.3 ± 0.2	2.2 ± 0.2^abc^	2.3 ± 0.1^e^	2.3 ± 0.2^f^	2.4 ± 0.1	<**0**.**001**
Albumin (g/dL)	3.9 ± 0.6	3.5 ± 0.5^abc^	4.0 ± 0.6^de^	3.8 ± 0.6^f^	4.4 ± 0.3	<**0**.**001**
Phosphate (mg/dL)	3.4 ± 0.7	3.5 ± 0.6	3.4 ± 0.7	3.4 ± 0.5	3.4 ± 0.5	0.65
Creatinine (mg/dL)	1 ± 0.9	1 ± 1.1^c^	1 ± 0.7^d^	1.2 ± 1.8^f^	0.8 ± 0.7	**0**.**01**
**Comorbidity-related variables**						
Hypertension	541 (52.5%)	90 (54.2%)	337 (58.6%)^e^	48 (55.8%)	66 (44.9%)	**0**.**03**
Diabetes mellitus	228 (23.4%)	47 (28.3%)^c^	138 (24.0%)^e^	23 (26.7%)^f^	20 (13.6%)	**0**.**01**
Heart disease	294 (30.2%)	45 (27.1%)	192 (33.4%)^e^	26 (30.2%)	31 (21.9%)	**0**.**03**
Liver disease	48 (4.9%)	6 (3.6%)	27 (4.7%)	5 (5.8%)	10 (6.8%)	0.59
Chronic kidney disease	81 (8.3%)	19 (11.4%)	52 (9.0%)	4 (4.7%)	6 (3.4%)	0.06
Thyroid disease	89 (9.1%)	7 (4.2%)^c^	51 (8.9%)^e^	5 (5.8%)^f^	26 (17.7%)	<**0**.**001**
Parathyroid gland disease	17 (1.7%)	0 (0%)^c^	9 (1.6%)	1 (1.2%)	7 (4.8%)	**0**.**01**
Osteoarthritis	281 (28.9%)	42 (25.3%)	177 (30.8%)	28 (32.6%)	34 (23.1%)	0.17
Psychotic disorders	224 (23.0%)	47 (28.3%)	130 (22.6%)	20 (23.3%)	27 (18.4%)	0.21
Neurologic disease	155 (15.9%)	45 (27.1%)^ac^	80 (13.9%)	20 (23.3%)^f^	10 (7.5%)	<**0**.**001**
Cancer	101 (10.4%)	25 (15.1%)	52 (9.0%)	10 (11.6%)	14 (9.5%)	0.15

^a^Significant difference from “Hip” and “Vertebral”.

^b^Significant difference from “Hip” and “Hip + Vertebral”.

^c^Significant difference from “Hip” and “No Hip and Vertebral”.

^d^Significant difference from “Vertebral” and “Hip + Vertebral”.

^e^Significant difference from “Vertebral” and “No Hip and Vertebral”.

^f^Significant difference from “Hip + Vertebral” and “No Hip and Vertebral”.

*There are 6 groups in the post-hoc test, so a significant p-value should be less than 0.05/6 = 0.0083.

HF: Patients with only hip fracture.

VF: Patients with only vertebral fracture.

HF + VF: Patients with both hip fracture and vertebral fracture.

NO HF/VF: Patients with neither hip fracture nor vertebral fracture.

### Outcomes

**Figure 2:**
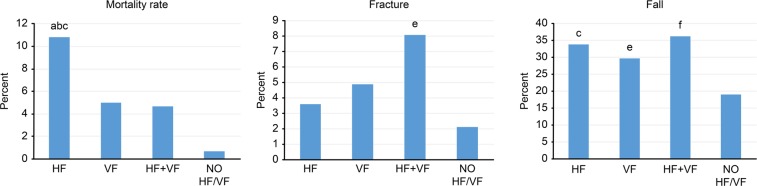
Primary outcomes of participants grouped according to fracture types. Post hoc analysis with Fisher’s least significant difference was applied to compare group difference. The P value was 0.0083 for significance group difference. Significant group differences were demonstrated alphabet. For mortality analysis, (**a**) significant difference from “HF” and “VF” (P = 0.0064); (**b**) significant difference from “HF” and “HF + VF” (P = 0.0073); (**c**) significant difference from “HF” and “NO HF/VF” (P < 0.001). For subsequent facture, (**e**) significant difference from “HF + VF” and “NO HF/VF” (P = 0.003). For fall, (**c**) significant difference from “HF” and “NO HF/VF” (P < 0.001); (**e**) significant difference from “VF” and “NO HF/VF” (P = 0.0053); (**f**) significant difference from “HF + VF” and “NO HF/VF” (P < 0.001). HF: Participants with hip fracture. VF: Participants with vertebral fracture. HF + VF: Participants with hip fracture and vertebral fracture. NO HF/VF: Participants with no hip fracture or vertebral fracture.

The one-year mortality rate was highest in the HF group and lowest in the NO HF/VF group (Fig. 1). In the post hoc analysis, mortality was significantly different for the HF vs. VF (10.8% vs. 5.0%, relative risk (RR) 2.15, 95% confidence interval (CI): 1.23–3.77, P < 0.01), HF vs. HF + VF (10.8% vs. 4.7%, RR 2.33, 95% CI: 0.95–6.76, P < 0.01) and HF vs. NO HF/VF (10.8% vs. 0.7%, RR 15.94, 95% CI: 2.15–117.94, P < 0.001) groups. For the rate of subsequent fractures, the HF + VF group had the highest rate, and the NO HF/VF group had the lowest rate over one year of follow-up. In the post hoc analysis, the rate of subsequent fractures was significantly different only between the HF + VF and NO HF/VF groups (8.1% vs. 2.1%, RR 3.99, 95% CI: 1.06–15.02, P < 0.003). Finally, the HF + VF group had the highest rate of falls, and the NO HF/VF group had the lowest rate of subsequent falls over one year of follow-up. In the post hoc analysis, the fall rate was significantly different between the HF + VF and NO HF/VF (36.1% vs. 19.0%, RR 1.89, 95% CI: 1.22–2.93, P < 0.001), HF and NO HF/VF (33.7% vs. 19.0%, RR 1.77, 95% CI: 1.19–2.63, P < 0.001), and the VF vs. NO HF/VF (29.6% vs. 19.0%, RR 1.55, 95% CI: 1.09–2.22, P < 0.0053) groups (Fig. 2).

**Figure 3:**
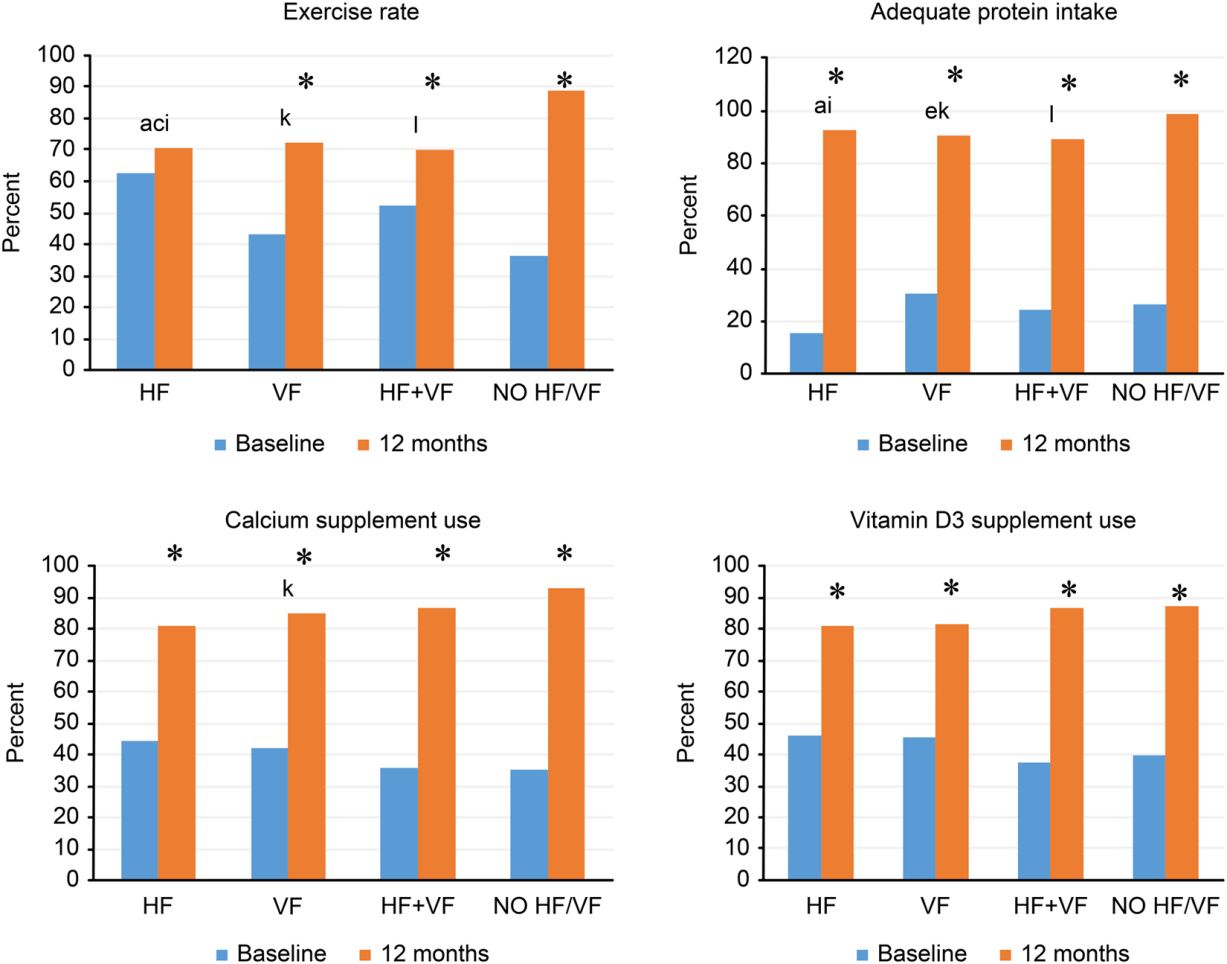
Secondary outcomes of participants grouped according to fracture types. *After intervention, there were significant difference between baseline and 12-month in exercise rate, adequate protein intake, calcium and vitamin D_3_ supplement use. Post hoc analysis with Fisher’s least significant difference was applied to compare group difference. The P value was 0.0083 for significance group difference. Significant between group difference was demonstrated by alphabet. For exercise rate of baseline assessment, a: significant difference from “HF” and “VF” (P < 0.001); c: significant difference from “HF” and “NO HF/VF” (P < 0.001). For exercise rate of 12-month assessment, i: significant difference from “HF” and “NO HF/VF” (P < 0.001); k: significant difference from “VF” and “NO HF/VF” (P < 0.001); l: significant difference from “HF + VF” and “NO HF/VF” (P < 0.001). For adequate protein intake of baseline assessment, a: significant difference from “HF” and “VF” (P < 0.001); e: significant difference from “VF” and “NO HF/VF” (P < 0.001). For adequate protein intake of 12-month assessment, i: significant difference from “HF” and “NO HF/VF” (P < 0.001); k: significant difference from “VF” and “NO HF/VF” (P < 0.001); l: means significant difference from “HF + VF” and “NO HF /VF” (P < 0.001). For calcium supplement use in 12-month assessment, k: significant difference from “VF” and “NO HF/VF” (P = 0.0065).

At baseline, for exercise habits, the HF group had the highest rate (HF vs. VF [62.7% vs. 43.3%, RR 1.45, 95% CI: 1.25–1.68, P < 0.001] and HF vs. NO HF/VF [62.7% vs. 36.1%, RR 1.74, 95% CI: 1.3–2.22, P < 0.001]) (Fig. 3). For adequate protein intake, the HF group had the lowest rate (HF vs. VF [15.7% vs. 30.8%, RR 0.51, 95% CI: 0.35–0.74, P < 0.001]). For calcium and vitamin D_3_ supplementation, there were no significant differences among the four groups. At 12 months, the NO HF/VF group had the highest rates of exercise habits (HF vs. NO HF/VF [70.7% vs. 89.0%, RR 0.80, 95% CI: 0.71–0.89, P < 0.001], VF vs. NO HF/VF [72.5% vs. 89.0%, RR 0.82, 95% CI: 0.76–0.89, P < 0.001], and HF + VF vs. NO HF/VF [70.2% vs. 89.0%, RR 0.79, 95% CI: 0.68–0.92, P < 0.001]) and adequate protein intake (HF vs. NO HF/VF [92.7% vs. 98.6%, RR 0.94, 95% CI: 0.90–0.99, P = 0.01], VF vs. NO HF/VF [90.3% vs. 98.6%, RR 0.92, 95% CI: 0.89–0.95, P < 0.001] and HF + VF vs. NO HF/VF [89.3% vs. 98.6%, RR 0.90, 95% CI: 0.83–0.97, P < 0.001]). There were no between-group differences in the use of calcium/vitamin D_3_ supplements. After intervention, adequate protein intake and the use of calcium/vitamin D_3_ supplementation were all increased significantly within each group over one year of follow-up (P < 0.001). However, for exercise habits, only the HF group had no significant within-group change from before to after the intervention.

### Regression analysis

Risk factors for higher mortality over one year of follow-up included older age, having HF or VF, having cancer, and steroid use. The HF group had the highest risk of mortality (odds ratio (OR) 6.78, 95% CI: 3.75–12.84) after adjusting for confounding factors (Table 2). Having a higher BMI and serum albumin level were associated with a lower risk of mortality. The HF + VF group was associated with a higher risk of incident fracture (OR = 4.25, 95% CI: 2.35–8.06) over one year of follow-up. Being older, having HF, VF, or HF + VF, and having cancer, osteoarthritis, or neurological disease were associated with a higher rate of falling over one year of follow-up. Having neurological disease was associated with the highest risk of falls (OR = 1.70, 95% CI: 1.13–3.82). Older patients have lower probability of regular exercise habits and adequate protein intake (OR = 0.98, 95% CI: 0.96–0.99 and OR = 0.94, 95% CI: 0.90–0.98, respectively). All patients having fractures have lower probability of regular exercise habits (HF: OR = 0.79, 95% CI: 0.74–0.84, VF: OR = 0.81, 95% CI: 0.77–0.87), and HF + VF (OR = 0.79, 95% CI: 0.74–0.84) and adequate protein intake HF (OR = 0.94, 95% CI: 0.89–0.98), VF (OR = 0.91, 95% CI: 0.90–0.95), and HF + VF (OR = 0.88, 95% CI: 0.84–0.94 ) than those without fracture. Patients having diabetes (OR = 1.86, 95% CI: 1.04–2.47), and psychosis (OR = 3.35, 95% CI: 2.66–4.14) were associated with higher probability of adequate protein intake, and those with lower level of serum albumin were associated with higher probability of adequate protein intake and vitamin D3 supplementation after intervention. Having liver disease was associated with lower probability of using calcium (OR = 0.56, 95% CI: 0.32–0.94) and vitamin D_3_ (OR = 0.33, 95% CI:0.27–0.89) supplement.

**Table 2:** Adjusted OR for selected outcomes by participant characteristics.

		Mortality OR (95% CI)	Incident Fracture OR (95% CI)	Fall OR (95% CI)	Exercise rate OR (95% CI)	Adequate protein intake OR (95% CI)	Calcium supplement use OR (95% CI)	Vitamin D supplement use OR (95% CI)
Age (years)		1.08*** (1.02–1.13)		1.02* (1.00–1.06)	0.98*** (0.96–0.99)	0.94*** (0.90–0.98)		
Fracture type (reference: NO HF/VF)	HF	6.78** (3.75–12.84)		1.36* (1.08–2.65)	0.80*** (0.75–0.85)	0.94** (0.89–0.98)		
	VF	3.67* (2.03–5.95)		1.32* (1.06–2.23)	0.81*** (0.77–0.87)	0.91*** (0.90–0.95)	0.91** (0.89–0.95)	
	HF + VF		4.25* (2.35–8.06)	1.61* (1.13–3.42)	0.79*** (0.74–0.84)	0.88*** (0.84–0.94)		
BMI (kg/m^2^) (reference: BMI > 21)		2.06* (1.22–3.76)						
Steroid use		3.70* (1.12–8.85)						
Rheumatoid arthritis							0.45* (0.20–0.96)	
ALP (U/L) (reference: ALP < 100)					0.76* (0.56–0.94)			
Albumin (g/dL) (reference: Alb > 3.8)		2.85* (1.58–5.39)				1.92* (1.14–2.55)		1.56* (1.12~2.16)
Diabetes						1.86* (1.04–2.47)		
Liver disease							0.56* (0.32–0.94)	0.33* (0.27–0.89)
Osteoarthritis				1.53** (1.08–2.67)				1.72* (1.28–2.25)
Psychosis						3.35* (2.66~4.14)		
Neurologic disease				1.70** (1.13–3.82)				
Cancer		2.13*** (1.32–4.00)		1.56* (1.11–2.33)				

CI: confidence interval; OR: odds ratio.

HF: Participants with hip fracture.

VF: Participants with vertebral fracture.

HF + VF: Participants with hip fracture and vertebral fracture.

NO HF/VF: Participants with no hip fracture or vertebral fracture.

*P < 0.05, **P < 0.01, ***P < 0.001.

## Discussion

There were significant differences among the four different fracture type groups at baseline. After one year of follow-up and intervention, the HF group still had the highest mortality rates, but the HF + VF group had the highest rates of subsequent fractures and incident falls. Other risk factors for mortality were steroid use and with previous history of cancer. Higher BMI and serum albumin levels were associated with a lower mortality rate. Patients with cancer, osteoarthritis and neurological diseases were associated with higher risk of incident fall. Most of the lifestyle factors that could be adapted for prevention of fragility fracture were improved after intervention in the within-group analysis.

The mortality rate after HF ranged from 10–45% in the first year, and the rates declined with time^14^. From the study using the Taiwan National Health Insurance database from 1999 to 2009, the annual mortality rate for patients with HF decreased from 18.1 to 14%^15^. In line with other studies evaluating FLS effects on mortality, participants with HF who were enrolled in an FLS had a lower mortality rate (10.8% for HF)^9,16^. The multifaceted interventions include regular assessment of adherence to medications and lifestyle modifications under a dedicated team, which seem to be a successful approach to improve patient outcomes^17^.

Patients are at a higher risk of subsequent fractures immediately after a sentinel osteoporotic fracture^18^. FLSs were established to reduce refracture^19^. Overall, the refracture rate (4.0%) was lower than that from the systemic review of data from five FLS studies with heterogeneous study designs (4.2–12.2%)^20^ and the Taiwan NHI data analysis (5.15%)^21^; nonetheless, only the HF + VF group had a higher refracture rate (8.1%). From a recent study using the Reykjavik Study fracture registration^22^, the risk of subsequent fracture was relatively low among those with a sentinel HF but increased among those with other sentinel fractures. Our results were in line with this study, showing the lowest risk among patients with HF but the highest risk for those with HF + VF. After controlling for confounders, having HF + VF was the only factor with a significantly higher risk of subsequent fractures. In previous clinical trials, patients without a prevalent fracture had a lower new fracture rate after pharmacological treatment for osteoporosis^23,24^. Similarly, in the current study, the NO HF/VF group had the lowest subsequent fracture rate after intervention. Refracture could be caused by poor muscle quality and posture alignments^25^; consequently, HF + VF, leading to insufficient muscle quality and postural misalignment, was associated with the highest risks of falls and refracture. Therefore, an individualized exercise program aiming for postural alignment correction and muscle quality strengthening should be established to prevent subsequent falls and refracture.

Malnutrition is a modifiable risk factor for functional loss, postoperative infection and poor healing of fractures^26^. In previous studies among Japanese patients^27,28^, hypoalbuminemia and low BMI were associated with higher mortality after newly diagnosed HF or VF. Similarly, we found that lower baseline albumin levels and lower BMI were associated with higher mortality, independent of fracture type and other risk factors. Malnutrition, which contributes to the loss of BMD, muscle quality and strength, could be a predictor for fragility fractures and subsequent adverse outcomes. Malnutrition is more prevalent when patients have some comorbidities, such as chronic kidney disease or liver disease because of dietary protein restriction based on the severity of these diseases. Individualized nutritional intervention should be integrated into multifaceted interventions to reduce fragility fractures and their complications.

Lifestyle modifications are also regarded as an essential part of intervention to prevent osteoporosis. Similar to the results of several previous studies^29–31^, most of our participants showed significant improvement in calcium/vitamin D_3_ intake, exercise habits and adequate protein intake^32,33^. However, both HF and VF groups have a greater impact on the ability to exercise, and only 70.7% of patients with HF had exercise habits after the one-year intervention. Also, older patients have lower rate of regular exercise. More aggressive strategies immediately after the fracture should be undertaken by those with newly developed fracture to prevent further functional decline.

There were several limitations of the current study. First, we only recorded new fractures with clinical evidence (defined as self-reported major osteoporotic fractures, including the hip, spine, wrist, and upper arm). Therefore, our refracture rate was lower than that of programs that include broader categories of fractures or longer follow-up^19,21^. The information about FRAX® scores, comorbidities, exercise habits, adequate protein intake, calcium and vitamin D_3_ intake were obtained from subjective yes-or-no question. Further objective tools to evaluate change of patients’ lifestyle factors after fracture liaison services were needed for qualification analysis. Second, we did not consider whether surgical interventions or postoperative complications of HF and VF have associations with one-year outcomes. Third, the sample size of each group had an uneven distribution, and the follow-up time was relatively short. Certain factors contributing to adverse outcomes might not be revealed in our study. Despite these limitations, our results would be considered to be generalized to clinical practice or cohort study worldwide with several merits. First, of our participants were similar to general population with diverse comorbidities. Second, we only excluded patients having fractures caused by major trauma, cancer, or an atypical fracture at the femoral shaft; and those with life expectancy of less than 2 years. Third, the dropout rate (<10%) and missing data (<5%) are relatively low. Fourth, we included demographic, laboratory data and comorbidities in logistic regression to reduce the influence of confounding factors.

In conclusion, the baseline characteristics and outcomes were different among the groups with different fracture types. Our services improved the outcomes and maintained the patients’ optimal lifestyles to prevent fragility fractures. Nonetheless, patients with HF had poor health status and still had the highest mortality rate of the patients enrolled in our services. Early initiation of multifaceted interventions is important to prevent fragility fractures and subsequent adverse outcomes among those with osteoporosis.

## Methods

### Study oversight

We conducted a prospective interventional study from 2014 to 2016 with two complementary parts: the fracture liaison service (FLS) and medication management service (MMS) programs at National Taiwan University Hospital (NTUH) main hospital (MH) and its Bei-hu branch (BB) which are all in Taipei, Taiwan. Both services were approved by the Research Ethics Committee at the National Taiwan University Hospital (NTUH) (FLS 201311048RINC, and MMS 201406077RINA). All participants provided written informed consents. We conducted FLS according to the 13 standards of the Best Practice Framework (BPF)^6,34^ to provide comprehensive care for patients with osteoporotic fractures. Complementary with FLS, patients taking osteoporosis medications for high fracture risk but not fitting the enrollment criteria of FLS were enrolled for MMS which was also conducted according to the 13 standards of BPF.

### Participants

#### Fracture liaison service (FLS)

Participants were recruited through inpatient departments and outpatient clinics and underwent comprehensive medical screening. At MH and BB of NTUH, patients who were aged 50 years or older and had one of the following 3 conditions were enrolled: (1) patients with newly developed HFs being hospitalized in orthopedic wards; (2) patients with newly identified VFs among those being hospitalized in geriatric wards; and (3) patients with clinical VFs who presented to outpatient clinics. Patients were excluded from these services if they had one of the following conditions: (1) fractures caused by major trauma, cancer, or an atypical fracture at the femoral shaft; (2) life expectancy of less than 2 years; (3) recruited for other fracture or osteoporosis intervention trials; and (4) unable or unwilling to complete assessments and follow-up visits according to the study protocol.

For inpatients in MH and BB, our team members identified patients who were hospitalized for HFs. To identify patients with untreated osteoporosis-related VFs, team members searched the imaging reports of patients in the geriatric ward. Subsequently, the coordinators provided initial assessments during their hospital stay after patients provided written informed consent. Patients who visited team physicians in outpatient clinics for symptoms of osteoporosis (loss of height, back pain, and kyphoscoliosis, etc.) had spine X-ray performed. When untreated VFs were found, coordinators provided initial assessments after patients provided written informed consent.

#### Medication management service (MMS)

The MMS enrolled patients who were 50 years of age or older at the outpatient clinics in MH and BB with any of the following conditions: (1) new initiation of osteoporosis medications; (2) change in osteoporosis medications; (3) poor compliance of osteoporosis medications; and (4) team physicians recommended that medication management might benefit the patients. Therefore, the differences between FLS and MMS were (1) patients without clinical evidence of osteoporotic fracture being prescribed osteoporosis medications and (2) new users of osteoporosis medications for patients with old fractures or when osteoporosis was not identified. The exclusion criteria were the same as those in FLS. FLS was prioritized when a patient fulfilled the enrollment criteria of both services.

Osteoporosis medications have been shown to reduce subsequent fractures and mortality after osteoporotic fractures^11,35^. Because the current study aimed to investigate whether fracture types affect outcomes, we needed to control this important determinant. Therefore, all participants entering the final analysis were prescribed osteoporosis medications after enrollment. For comparison of the outcomes among participants having different fracture types, we regrouped them within the FLS and MMS programs into 4 groups according to fracture types, namely, hip fracture (HF), vertebral fracture (VF), hip plus vertebral fracture (HF + VF) and no hip or vertebral fracture (NO HF/VF).

### Study outcomes

The primary outcomes, including subsequent fracture rate, fall rate, and mortality, within a one-year follow-up were examined among groups. The secondary outcomes were changes in the use of calcium/vitamin D_3_ supplements, the rate of regular exercise, and adequate protein intake (>65 g/day), and these were assessed within groups and among groups. The rate of regular exercise (30 minutes of exercise each time), use of calcium/vitamin D_3_ supplement, and adequate protein intake (more than 65 grams of protein per day) were also obtained by self-reported yes or no questions through telephone interviews or follow-up clinic visit.

### Intervention

Coordinators gave participants study brochures for education on preventing osteoporosis, falls and subsequent fractures and asked the participants about their subjective understanding of the brochures. Participants who were at high risk for subsequent falls were referred to team geriatricians for detailed assessments, including medical history, medication assessments, and focused physical and mental examinations. According to the results of the detailed assessment, interventions were planned, such as medication reconciliation or rehabilitation.

### Baseline assessments

Collected information including demographics, osteoporosis/fracture/fall history, life styles to prevent osteoporosis (regular exercise; use of calcium and vitamin D_3_ supplements, adequate protein intake), and self-reported comorbidities (including hypertension, diabetes, heart disease, liver disease, chronic kidney disease [CKD], thyroid disease, parathyroid gland disease, rheumatoid arthritis, osteoarthritis, cancer, neurological and psychotic disorders) was obtained by questionnaire. BMD data by DXA which were obtained in clinic visit after enrollment, and current osteoporosis medications were identified from medical records at MH and BB. Other determinants FRAX® including steroid use (yes- if the patients use oral steroid currently or have used oral steroid for more than 3 months; the dose of prednisolone of 5 mg daily or more [or equivalent doses of other steroid]) were obtained by question. Laboratory data (serum calcium, albumin, phosphate, and creatinine levels) were obtained to exclude secondary osteoporosis.

### Follow-Up assessments

After baseline assessments, coordinators called the participants to complete their regular clinic visits, and telephone interviews were performed at 4, 8, and 12 months. The collected information included interim death, falls, subsequent fractures, medications and calcium/vitamin D_3_ supplement adherence, side effects of medications, changes in indication, and rate of exercise.

### Statistical analysis

Baseline characteristics were compared with the use of analysis of variance or chi-square test with Fisher’s exact test. When the overall P value for the interaction among groups was less than 0.05, post hoc analysis with Fisher’s least significant difference was applied to compare group differences and the P value was presented accordingly. Relative risk including 95% confidence intervals for between group comparison using chi-square test with Fisher's exact test was also presented. Missing data was less than 5% and the expectation-maximization (EM) algorithm was used for incomplete data.

Longitudinal changes from before to after intervention within groups were tested with paired-t tests and McNemar’s tests. Multivariate logistic stepwise regression models were used to identify baseline factors associated with the development of each outcome of interest at one year. Baseline factors included age, sex, FRAX related variables, health-related variables, and comorbidity-related variables. Age, sex and FRAX-related variables were important confounding factors for our primary and secondary outcomes. The health-related variables were considered as important representative presentations of secondary osteoporosis. Important comorbidities were selected based on prior knowledge. All statistical analyses were conducted using SPSS software, version 22.0 (SPSS Inc, Chicago, IL).

## Data Availability

Data are available upon request to corresponding author when indicated.
